# Neutrophils Expressing Programmed Death-Ligand 1 Play an Indispensable Role in Effective Bacterial Elimination and Resolving Inflammation in Methicillin-Resistant *Staphylococcus aureus* Infection

**DOI:** 10.3390/pathogens13050401

**Published:** 2024-05-11

**Authors:** Azusa Terasaki, Faizan Ahmed, Alato Okuno, Zhenzi Peng, Duo-Yao Cao, Suguru Saito

**Affiliations:** 1 Department of Breast-Thyroid-Endocrine Surgery, University of Tsukuba, Ibaraki 3058577, Japan; terasaki.azusa.gf@un.tsukuba.ac.jp; 2 Division of Gastroenterology, Cincinnati Children’s Hospital Medical Center, Cincinnati, OH 45229, USA; Faizan.Ahmed@cchmc.org; 3 Department of Health and Nutrition, Faculty of Human Design, Shibata Gakuen University, Aomori 0368530, Japan; a-okuno@shibata.ac.jp; 4 Department of Cell Biology and Genetics, School of Basic Medical Sciences, Hengyang Medical School, University of South China, Hengyang, Hunan 421001, China; zzpivy@usc.edu.cn; 5 Department of Biomedical Sciences, Cedars-Sinai Medical Center, Los Angeles, CA 90048, USA; 6 Division of Virology, Department of Infection and Immunity, Faculty of Medicine, Jichi Medical University, Tochigi 3290431, Japan

**Keywords:** PD-L1, neutrophil, MRSA, PD-1, immunosuppressive

## Abstract

Programmed death ligand 1 (PD-L1) is a co-inhibitory molecule expressed on the surface of various cell types and known for its suppressive effect on T cells through its interaction with PD-1. Neutrophils also express PD-L1, and its expression is elevated in specific situations; however, the immunobiological role of PD-L1^+^ neutrophils has not been fully characterized. Here, we report that PD-L1-expressing neutrophils increased in methicillin-resistant *Staphylococcus aureus* (MRSA) infection are highly functional in bacterial elimination and supporting inflammatory resolution. The frequency of PD-L1^+^ neutrophils was dramatically increased in MRSA-infected mice, and this population exhibited enhanced activity in bacterial elimination compared to PD-L1- neutrophils. The administration of PD-L1 monoclonal antibody did not impair PD-L1^+^ neutrophil function, suggesting that PD-L1 expression itself does not influence neutrophil activity. However, PD-1/PD-L1 blockade significantly delayed liver inflammation resolution in MRSA-infected mice, as indicated by their increased plasma alanine transaminase (ALT) levels and frequencies of inflammatory leukocytes in the liver, implying that neutrophil PD-L1 suppresses the inflammatory response of these cells during the acute phase of MRSA infection. Our results reveal that elevated PD-L1 expression can be a marker for the enhanced anti-bacterial function of neutrophils. Moreover, PD-L1^+^ neutrophils are an indispensable population attenuating inflammatory leukocyte activities, assisting in a smooth transition into the resolution phase in MRSA infection.

## 1. Introduction

Neutrophils play a crucial role in innate defense against various pathogens [1]. They are abundantly present in the bloodstream and are continuously produced by bone marrow (BM), allowing for rapid and abundant recruitment to sites of infection, where they contribute to pathogen elimination [2]. Neutrophils employ a variety of mechanisms, including myeloperoxidase (MPO), neutrophil elastase (NE), neutrophil extracellular trap (NET), superoxide and cytokine productions, as well as phagocytosis, to aggressively eliminate pathogens through these physical and chemical-mediated strategies [3]. While neutrophils have long been understood as a homogeneous population, recent studies have revealed that they are a heterogeneous population, particularly in abnormal conditions, such as infectious diseases and cancers [4,5]. Therefore, characterizing each neutrophil population and their functions have been necessary to understand the role of neutrophils in specific situations.

Recent studies have shown that neutrophils expressing programmed death-ligand 1 (PD-L1, also known as CD274) are immunosuppressors in cancers [5,6]. PD-L1 is a well-known suppressive molecule expressed on tumor cells and some myeloid cells in the tumor microenvironment (TME), and PD-L1^+^ myeloid cells are often observed even in the peripheral tissues and organs [7,8,9]. In the context of tumor immunology, PD-L1-expressing cells suppress T cell function by interacting directly with PD-1 [10]. This interaction triggers the phosphorylation of the immunoreceptor tyrosine-based switch motif (ITSM) in the cytosolic tail of PD-1, recruiting SH2 domain-containing protein tyrosine phosphatase-2 (SHP-2), which results in the dephosphorylation of zeta-chain-associated protein kinase 70 (ZAP70) in T cells, thereby suppressing their functions [11,12]. This mechanism of functional suppression is widely conserved in PD-L1-expressing cells; therefore, PD-L1^+^ neutrophils are mostly considered to participate in an immunosuppressive role in cancer. However, other functional characterizations of PD-L1^+^ neutrophils, especially in non-cancer conditions, are still insufficient.

In this report, we demonstrate that PD-L1^+^ neutrophils exhibit higher immune activities than PD-L1^−^ neutrophils, which is characterized by their predominant role for bacterial elimination in MRSA-challenged mice. Moreover, PD-L1^+^ neutrophils play an important role in maintaining the inflammatory resolution mechanism during the acute phase of MRSA infection. Our finding provides a novel understanding of the immunological role of PD-L1^+^ neutrophils in bacterial infection and acute inflammation.

## 2. Materials and Methods

*Reagents and antibodies*. Anti-PD-L1 (10F.9G2), anti-CD45 (30-F11), anti-CD11b (M1/70), anti-Ly-6G (1A8), anti-Ly-6C (HK1.4), anti-F4/80 (BM8), anti-CD3 (17A2), anti-CD4 (GK1.5), anti-CD8 (53–6.7), anti-TCRγδ (UC7-13D5), anti-NK1.1 (S17016D), anti-IFN-γ (XMG1.2), anti-IL-17A (TC11-18H10.1), anti-IL-6 (MP5-20F3), and anti-TNF-α (MP6-XT22) were purchased from BioLegend (San Diego, CA, USA). Anti-IL-1β (EPR24895-116), anti-myeloperoxidase (MPO, EPR20257), and anti-neutrophil elastase (NE, EPR7479) were purchased from Abcam (Cambridge, UK). Anti-granzyme B (NGZB), anti-perfolin (eBioOMAK-D), 2′,7′-dichlorodihydrofluorescein diacetate (H_2_DCFDA), SYTOX^TM^ Green, 7-amino-actinomycin D (7-AAD), and fluorescein isothiocyanate (FITC)-labeled *Staphylococcus aureus* (SA-FITC) were purchased from Thermo Fisher Scientific (Waltham, MA, USA). Anti-PD-L1 mAb (10F.9G2) and its corresponding isotype antibody were purchased from BioXcell (Lebanon, NH, USA). Phorbol-12-myristate-13-acetate (PMA), ionomycin, and an ALT activity assay kit were purchased from Sigma Aldrich (Laclede Avenue. St. Louis, MO, USA). Golgi Stop^TM^ was purchased from BD Bioscience (Franklin Lakes, NJ, USA). Percoll was purchased from GE HealthCare (Chicago, IL, USA). Recombinant murine granulocyte-macrophage colony-stimulating factor (rmGM-CSF) was purchased from Peprotech (Cranbury, NJ, USA).

*Methicillin-resistant Staphylococcus aureus culture and sample preparation.* The frozen *S. aureus* (MRSA; USA300) stock was thawed on ice, and then transferred to a tryptic soy broth (TSB; BD Bioscience, Franklin Lakes, NJ, USA). The bacterial suspension was cultured at 37 °C for 18 h with shaking, and then the grown bacteria were further cultured in TSB at a 1:100 dilution at 37 °C for 6–8 h. The colony forming unit (CFU) was calculated by the standard curve method for each culture. For heat-killed *S. aureus* (HK-SA) preparation, the bacterial suspension was heated at 95 °C for 15 min. The heated *S. aureus* suspension was centrifuged at 10,000 rpm for 1 min to harvest the bacteria cells and washed with phosphate-buffered saline (PBS), and then the cell pellet was resuspended in PBS. The cell wall extract (CWE), crude protein extract (CPE), lipoprotein (LP), and nucleic acids were isolated from the *S. aureus* by following a method described in previous reports, with minor modifications [13,14]. Briefly, the cultured *S. aureus* (1.0 × 10^8–9^ CFU/mL) was harvested by centrifugation at 5000× *g* for 20 min, and then the pellet was washed twice with Tris-HCl (20 mM, pH 8.0). The pellet was resuspended in Tris–HCl (20 mM, pH 8.0) and stored at −80 °C overnight. The sample was centrifuged at 5000× *g* for 20 min again, and then the bacterial cells were crushed with 0.3 mm stainless beads, followed by sonication at 4 °C for 20 min. The treated sample was centrifuged at 5000× *g* for 20 min, and then the supernatant was collected as containing proteins. The precipitated pellet was treated with protease (1 μg/mL) and lipoprotein lipase (1 μg/mL) at 37 °C for 1 h, followed by heating at 95 °C for 15 min. The sample was washed with Tris–HCl (20 mM, pH 8.0) and centrifuged at 5000× *g* for 20 min. The precipitated sample was used as the CWE. The supernatant containing protein was mixed with an equal volume of 100% ethanol and kept at −80 °C overnight. The sample was centrifuged at 12,000× *g* for 15 min, and then the precipitated pellet was washed with 80% ethanol and centrifuged again at 12,000× *g* for 15 min. The precipitated pellet was dissolved with 1 M urea/50 mM Tris-HCl and 50 mM ethylenediaminetetraacetic acid (EDTA) (pH 8.0) and treated with DNase (50 μg/mL) and RNase (50 μg/mL) at 37 °C for 1 h, and then the sample was used as the CPE. For LP isolation, Triton X-114 was added to the CPE (final 1%), and then the suspension was incubated at 4 °C with gentle mixing. The sample was further incubated at 37 °C to form the micelle phase-containing LP. The micelle phase was collected, and the LP was precipitated by following a method for CPE precipitation. The precipitated pellet was used as the LP. The DNA and RNA were isolated from the bacteria using NucleoSpin^®^ (Thermo Fisher Scientific, Waltham, MA, USA) and TRIzol^®^ (Thermo Fisher Scientific), respectively.

*Animal experiment.* C57BL/6 mice were purchased from CLEA Japan (Tokyo, Japan) and The Jackson Laboratory (Bar Harbor, ME, USA). TLR2-KO mice (B6.129-*Tlr2^tm1Kir^*/J), TLR4-KO mice (B6(Cg)-*Tlr4^tm1.2Karp^*/J), and MyD88-KO mice (B6.129P2(SJL)-*Myd88^tm1.1Defr^*/J) were purchased from The Jackson Laboratory. All mice were bred in-house and maintained in a specific pathogen-free (SPF) facility with 12-h light/dark cycles and allowed free access to food and water. Adult mice of both genders aged 8–20 weeks were used for each experiment. All animal experimental protocols were approved by the Animal Care and Use Committee of Jichi Medical University (20038-01), University of South China (202005053) and Shibata Gakuen University (2107).

*MRSA infection*. MRSA infection was performed following a method described in previous report, with minor modification [14]. Briefly, MRSA was washed and resuspended in PBS at a 1.0 × 10^9^ CFU/mL concentration. The mice received an intravenous (i.v.) injection of MRSA (100 µL of suspension) through the retro orbital sinus. For in vivo PD-1/PD-L1 blockade, the MRSA-challenged mice were i.v. injected an isotype antibody or anti-PD-L1 mAb (200 µg in 100 µL of saline). The blood samples and tissue samples (spleen, liver, and lung) were collected at 48 h post-infection. Before organ extraction, the mice were perfused with PBS. The tissues were washed with PBS, then chopped and homogenized in PBS. The processed tissue samples were filtered through a 70 μm strainer and centrifuged at 300× *g* for 5 min. The supernatants were collected and serially diluted with PBS prior to seeding on TSB plates. The plates were incubated at 37 °C overnight, and then the MRAS CFUs were determined in the samples.

*Primary cell isolation.* Peripheral blood was collected using an ethylenediaminetetraacetic acid (EDTA)-2Na-coated tube and treated with red blood cell (RBC) lysis buffer at room temperature (RT) for 10 min. The sample was washed with PBS, and the leukocytes were collected by centrifugation at 300× *g* for 5 min. The spleen and lung were mechanically crushed on a 70 µm cell strainer in RPMI complete medium, and then the cells were collected by centrifugation at 300× *g* for 5 min. The cells were treated with RBC lysis buffer at RT for 10 min, and then washed with RPMI complete medium. After centrifugation at 300× *g* for 5 min, the precipitated cells were used as splenocytes and lung-isolated cells, respectively. Bone marrow (BM) cells were isolated from the femurs and tibias. The cells were flushed from the bones using a 10 mL syringe with a 27G needle in RPMI complete medium. The cells were collected by centrifugation at 300× *g* for 5 min, and then treated with RBC lysis buffer at RT for 10 min. After washing with PBS, the cells were collected by centrifugation at 300× *g* for 5 min. The precipitated cells were used as BM-isolated cells. Neutrophils were enriched from BM-isolated cells using the Neutrophil Isolation Kit, mouse (Miltenyi Biotec, Bergisch Gladbach, North Rhine-Westphalia, Germany). Hepatic leukocytes were isolated from the whole liver. The liver was excised from a PBS-perfused mouse, chopped, and mechanically crushed on a 70 µm cell strainer in RPMI complete medium, followed by digestion with collagenase (1 mg/mL) at 37 °C for 30 min. The samples were re-filtrated by passing through a 70 µm cell strainer, and the cells were collected by centrifugation at 300× *g* for 5 min. The precipitated cells were re-suspended in 35% Percoll (diluted with PBS) for density gradient separation by centrifugation at 600× *g* for 20 min without acceleration or braking. After centrifugation, the precipitated cells were further treated with RBC lysis buffer at RT for 10 min. The samples were washed with RPMI complete medium and centrifuged at 300× *g* for 5 min. The precipitated cells were used as hepatic leukocytes. Splenic CD4^+^ and CD8^+^ T cells were isolated from the splenocytes using the CD4^+^ T Cell Isolation Kit, mouse (Miltenyi Biotec, Bergisch Gladbach, North Rhine-Westphalia, Germany) and the CD8a^+^ T Cell Isolation Kit, mouse (Miltenyi Biotec), respectively. The isolation procedures were performed by following the product manuals. The purity of the neutrophils, CD4^+^, and CD8^+^ T cells was assessed by flow cytometry, and samples with more than 90% CD11b^+^Ly-6G^+^, CD4^+^, or CD8^+^ T cells were used for subsequent experiments.

*Flow cytometry and cell sorting.* Flow cytometry analysis was performed using the LSRII (BD Bioscience, Franklin Lakes, NJ, USA) and cell sorting was performed using the FACS Aria II (BD Bioscience). For extracellular marker staining, the samples were stained with fluorochrome-conjugated mAb or tetramer in the presence of anti-CD16/CD32 mAb for the blocking of Fc gamma Receptor (FcγR) II/III at 4 °C for 30 min. Intracellular staining was performed using the BD Cytofix/Cytoperm™ Fixation/Permeabilization Kit (BD Bioscience). For intracellular staining, the extracellular-stained cells were fixed at 4 °C for 20 min, followed by staining of the intracellular targets at 4 °C for 30 min. The samples studied for IL-1β production in neutrophils were treated with GolgiStop^TM^ (1 μg/mL) during the in vitro reaction. The samples studied for cytokine production in T cells were stimulated with PMA (100 ng/mL) and ionomycin (250 ng/mL) in the presence of GolgiStop^TM^ (1 μg/mL) at 37 °C for 5 h. ROS production was measured by staining with H_2_DCFDA (5 μM) at 37 °C for 30 min. The data were analyzed by the FlowJo 10 (BD Bioscience).

*Real-Time Polymerase Chain Reaction (Real-time PCR)*. The total RNA was isolated from neutrophils using TRizol (Thermo Fisher Scientific). The concentration and purity of the RNA were measured by NanoDrop 2000c (Thermo Fisher Scientific). The total RNA (250–500 ng) was used for reverse transcription for making complementary DNA (cDNA) using the PrimeScript™ RT-PCR Kit (Takara, Tokyo, Japan). The cDNA was used for quantitative PCR performed using a Thermal Cycler Dice^®^ Real Time System III (Takara, Tokyou, Japan). The mRNA expressions were quantified by the ΔCt method. The primer sequences used in the assay are represented in Supplementary Table S1.

*Culture for generating PD-L1^+^ neutrophils.* BM-isolated cells (3.0–5.0 × 10^6^/mL) were culture with RPMI complete medium supplemented with GM-CSF (100 ng/mL) at 37 °C for 24 h. The PD-L1^−^ and PD-L1^+^ cells were isolated from the CD11b^+^Ly-6G^+^ population in the pre-cultured BM cells by a cell sorter.

*Phagocytosis assay.* BM-isolated neutrophils (1.0 × 10^7^/mL) were incubated with FITC-labeled S. aureus (SA-FITC; 25 µg/mL) in RPMI complete medium at 37 °C for 2 h. The cells were washed with PBS and analyzed by flow cytometry. The fluorescence signal (mean fluorescence intensity; MFI) originating from the intracellular incorporated bacteria was used for assessing the phagocytosis activity in the neutrophils.

*Neutrophil stimulation.* BM-isolated neutrophils (1.0 × 10^7^/mL) were seeded in 96-well plates with RPMI complete medium. For the study of PD-L1 upregulation, the cells were treated with vehicle (PBS), HK-SA, CWE, CPE, LP, DNA, or RNA at their indicated concentrations at 37 °C for 6 h. After incubation, the cells were subjected to flow cytometry. Alternatively, the neutrophils were cultured at 37 °C for 3 h in total RNA isolation. For functional studies, the neutrophils were treated with PBS or HK-SA (1.0 × 10^8^ CFU/mL, MOI = 1:10) at 37 °C for 6 h, and then the cells were subjected to flow cytometry assay. For the IL-1β production assay, the neutrophils were cultured with PBS or HK-SA (the same MOI as the functional studies) at 37 °C for 18 h, and then the plate was immediately frozen at −80 °C. The plate was centrifuged at 300× *g* for 5 min, and the supernatant was subjected to IL-1β ELISA.

*In vitro MRSA killing.* Neutrophils (1.0 × 10^7^/mL) were mixed with MRSA (1.0 × 10^8^ CFU/mL, MOI = 1:10) in RPMI complete medium. The samples were incubated at 37 °C for 5 h. The samples were centrifuged at 300× *g* for 5 min, and then the supernatants were discarded, and the precipitated cells were washed with PBS. The cells were again collected by centrifugation at 300× *g* for 5 min, and then treated with PBS/0.1% Triton X-100 at RT for 15 min for lysing the cells. The supernatant samples were used for the determination of the MRSA CFUs by seeding on a TSB agar plate. The plates were incubated at 37 °C overnight, and then the MRAS CFUs were determined in each sample.

Neutrophils and T cell coculture. Neutrophils (2.5 × 10^6^/mL) and CD4^+^ or CD8^+^ T cells (2.5 × 10^6^/mL) (at a ratio = 1:1) were co-cultured in the presence of anti-CD3 mAb (10 µg/mL) and anti-CD28 mAb (2 µg/mL) at 37 °C for 24 h. For PD-1/PD-L1 blockade, the co-cultures were further treated with anti-PD-L1 mAb (10 µg/mL) or isotype antibody (10 µg/mL). In the last 5 h, the cells were stimulated with PMA (100 ng/mL) and ionomycin (250 ng/mL) in the presence of GolgiStop^TM^ (1 µg/mL). The IFN-γ production in the CD4^+^ and CD8^+^ T cells was analyzed by flow cytometry.

*Enzyme-linked immunosorbent assay (ELISA).* The mouse IL-1 beta/IL-1F2 DuoSet ELISA kit was purchased from R&D Systems (Minneapolis, MN, USA). All procedures followed the manufacturer’s instructions.

*Statistics.* Student’s *t*-test and one-way analysis of variance (ANOVA) were used to analyze the data for significant differences. Values of *p* < 0.05, *p* < 0.01, and *p* < 0.001 were regarded as significant.

## 3. Results

### 3.1. MRSA Infection Increases PD-L1 Expression in Neutrophils

**Figure 1:**
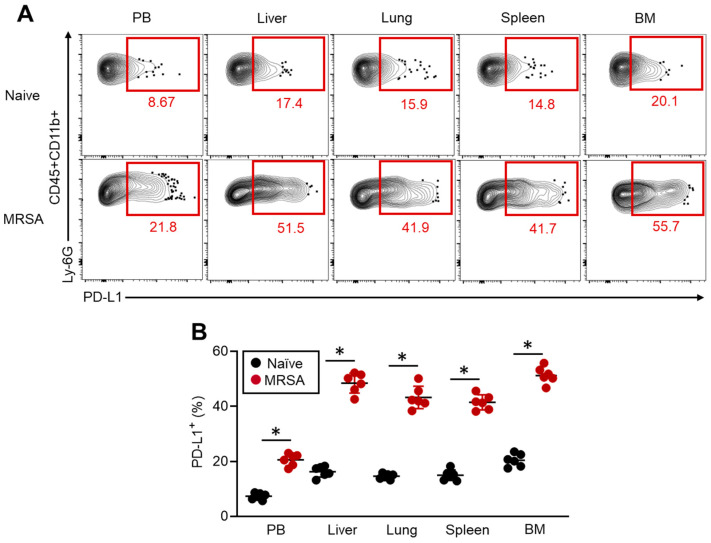
PD-L1 expression is increased in the neutrophils of MRSA-infected mice. The mice were infected to MRSA by i.v. injection, and the PD-L1 expression in neutrophils was analyzed in the PB, liver, lung, spleen, and BM at 48 h post-infection. Representative plots (**A**) and cumulative percentages of PD-L1^+^ neutrophils (**B**) are shown. The cumulative data are shown as the mean ± standard error (SD) of six samples. Student’s *t*-test was used to analyze the data for significant differences. Values of * *p* < 0.001 are regarded as significant.

We first investigated how PD-L1 expression changes in response to pathogenic invasion by establishing a murine MRSA infection model. The mice were intravenously infected with MRSA, and the cell surface expressions of PD-L1 in the neutrophils from the peripheral blood (PB), liver, lung, spleen and BM were analyzed by flow cytometry at 48 h post-infection. In naïve mice, PD-L1 was already expressed in some neutrophils, while the frequency of PD-L1^+^ neutrophils was less than or around 20% in each sample (PB: 7.312 ± 1.178; liver: 16.25 ± 1.937; lung: 14.58 ± 0.999; spleen: 14.98 ± 1.915; and BM: 20.42 ± 2.340) (Figure 1A, top; B, black dots). PD-L1 expression was significantly increased in the neutrophils from the MRSA-challenged mice. The neutrophils in all samples consistently exhibited around 2.5- to 3-fold increases in the frequencies of PD-L1^+^ cells compared to the naïve status mice (PB: 20.57 ± 2.189; liver: 48.47 ± 3.640; lung: 43.25 ± 4.071; spleen: 41.48 ± 2.736; and BM: 51.37 ± 3.035) (Figure 1A, bottom; B, red dots). Interestingly, the increase in PD-L1^+^ neutrophils was more pronounced in the liver, lung, spleen and BM compared to the PB. PD-L1 mRNA expression was also analyzed in the PB neutrophils from naïve and MRSA-challenged mice. Consistent with the protein expression, the PD-L1 mRNA expression was significantly elevated in the PB neutrophils isolated from the MRSA-challenged mice compared to that of the naïve mice (Figure S1). Therefore, MRSA infection upregulates PD-L1 expression in neutrophils.

### 3.2. PD-L1-Expressing Neutrophils Possess a Predominant Activity Contributing to Effective MRSA Elimination

**Figure 2:**
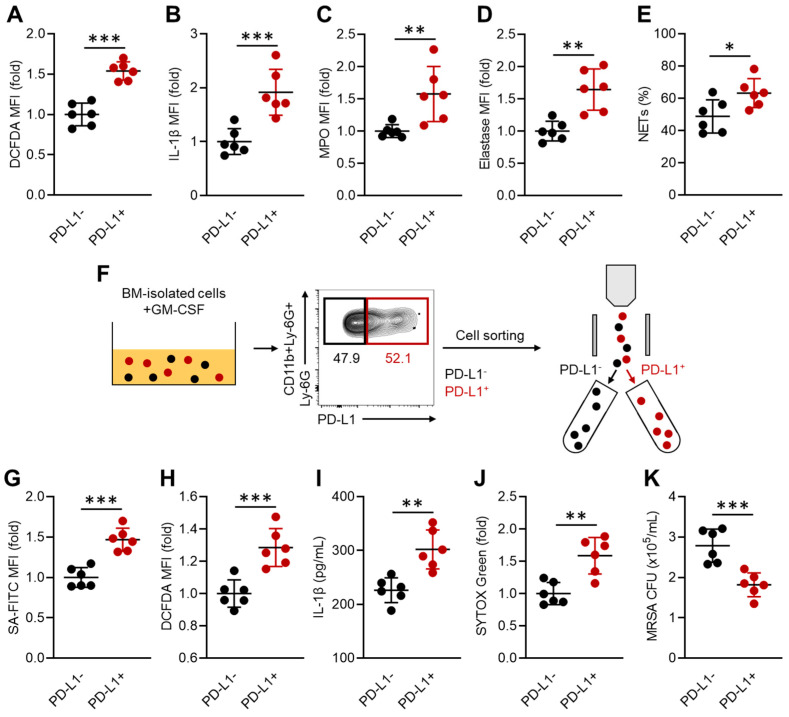
PD-L1^+^ neutrophils have enhanced anti-bacterial activity. (**A**–**E**) In vivo functional assay of neutrophils following PD-L1 expression. The activities were measured in the liver neutrophils 48 h after MRSA challenge. The productions of ROS (**A**), IL-1β (**B**), and MPO (**C**), and neutrophil elastase (**D**), and NET formation (**E**) were measured by flow cytometry. (**F**–**K**) In vitro functional assay of neutrophils. (**F**) The diagram of in vitro PD-L1 induction in neutrophils and sorting of PD-L1^+^ and PD-L1^−^ neutrophils. (**G**) Phagocytic activity against *S. aureus* (FITC-labeled). ROS (**H**) and IL-1β production (**I**) in HK-SA (MOI = 1:50)-stimulated neutrophils. (**J**) Cell-free-DNA (CFD) levels measured in the cultured medium of HK-SA (MOI—1:50)-stimulated neutrophils. (**K**) In vitro MRSA killing assay. Survival intracellular MRSA was quantified by measuring the CFU. The cumulative data are shown as the mean ± SD of six samples. Student’s *t*-test was used to analyze the data for significant differences. Values of * *p* < 0.05, ** *p* < 0.01, and *** *p* < 0.001 are regarded as significant.

Next, we investigated the function of PD-L1^+^ neutrophils in MRSA infection. Neutrophil activities were assessed in the livers of MRSA-infected mice at 48 h post-bacterial challenge following their PD-L1 expression status. Intriguingly, all the measured activities of neutrophils, including reactive oxygen species (ROS), interleukin (IL)-1β, MPO, NE production, and NET formation, were significantly increased in the PD-L1^+^ neutrophils compared to the PD-L1^−^ cells (Figure 2A–E). These findings were further investigated by in vitro experiments using neutrophils with artificially induced PD-L1 expression as the model cells. To do so, whole BM-isolated cells were cultured with GM-CSF at 37 °C for 24 h, which enabled us to obtain a sufficient number of PD-L1^+^ neutrophils. Subsequently, PD-L1^−^ and PD-L1^+^ neutrophils in the CD11b^+^Ly-6G^+^ population were respectively sorted by a cell sorter and subjected to each experiment (Figure 2F). In a phagocytosis assay using FITC-labeled *S. aureus* (SA), the PD-L1^+^ neutrophils captured more bacteria than the PD-L1^−^ cells (Figure 2G). Additionally, ROS production, IL-1β production, and NET formation under heat-killed SA (HK-SA) stimulation were all significantly increased in the PD-L1^+^ neutrophils compared to the PD-L1^−^ neutrophils (Figure 2H–J). Moreover, the PD-L1^+^ neutrophils exhibited greater efficacy in eliminating live MRSA than the PD-L1^−^ neutrophils in an in vitro bacterial killing assay (Figure 2K). Therefore, PD-L1^+^ neutrophils have increased anti-bacterial activity, which may serve as a major effector cell for bacterial elimination in MRSA infection.

### 3.3. MRSA Recognition via TLRs Increases PD-L1 Expression in Neutrophils

**Figure 3:**
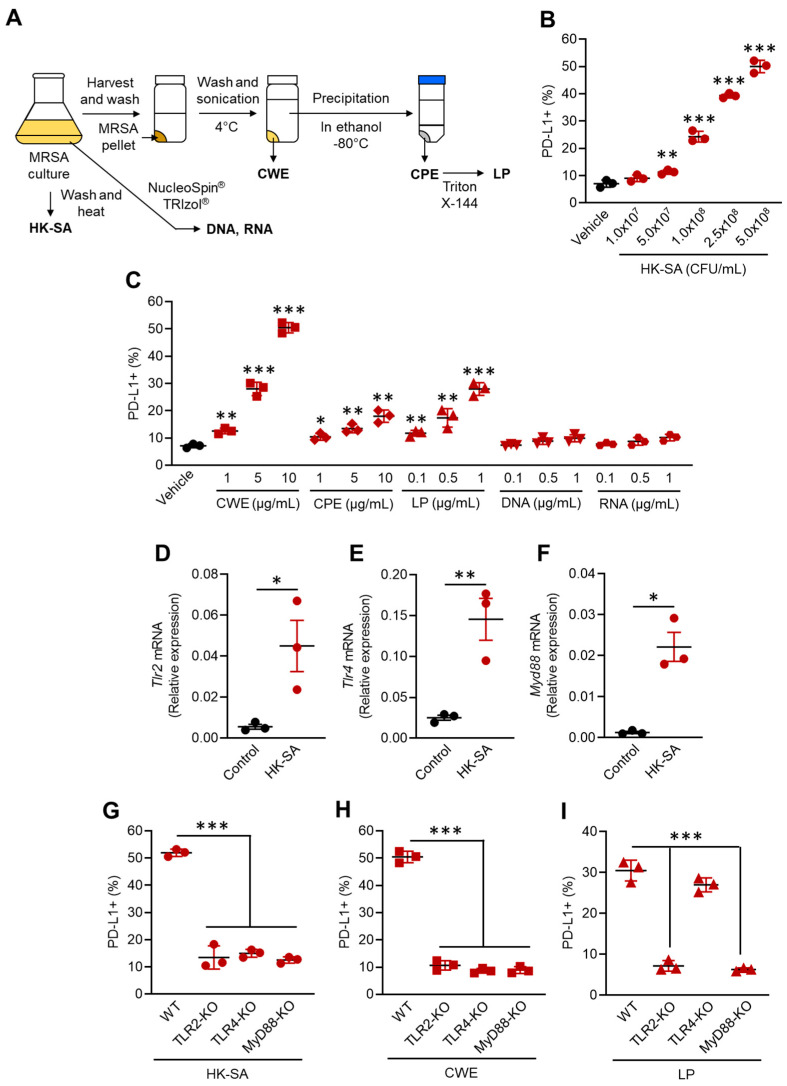
Bacterial structural components trigger PD-L1 expression in neutrophils through extracellular TLRs. (**A**) A diagram of the purification procedure of the MRSA structural components. (**B,C**) In vitro neutrophil stimulation. Neutrophils were isolated from WT mice BM and subjected to stimulation assay to measure the PD-L1 upregulation. (**B**) The percentages of PD-L1^+^ cells in the neutrophils cultured with vehicle (PBS) or HK-SA. (**C**) The percentages of PD-L1^+^ cells in the neutrophils cultured with vehicle (PBS), CWE, CPE, LP, DNA, or RNA isolated from MRSA. The mRNA expressions of TLR2 (**D**), TLR4 (**E**), and MyD88 (**F**) in vehicle (PBS) or HK-C60 (MOI = 1:50) cultured neutrophils. The percentages of PD-L1^+^ neutrophils cultured with HK-SA (MOI = 1:50) (**G**), CWE (10 μg/mL) (**H**), or LP (1 μg/mL) (**I**). The neutrophils were isolated from WT, TLR2-KO, TLR4-KO, or MyD88-KO mice. The cumulative data are shown as the mean ± SD of six samples. One-way ANOVA was used to analyze the data for significant differences. Values of * *p* < 0.05, ** *p* < 0.01, and *** *p* < 0.001 are regarded as significant.

We investigated the crucial mechanism involved in PD-L1 upregulation in neutrophils during MRSA infection. For this purpose, we prepared HK-SA and isolated structural components, such as cell wall extract (CWE), crude protein extract (CPE), lipoprotein (LP), and nucleic acids (DNA, RNA), from MRSA culture following the represented procedures based on previous publications (Figure 3A) [13,15]. In vitro HK-SA stimulation showed that PD-L1 expression was significantly increased in the neutrophils treated with HK-SA at concentrations greater than 5.0 × 10^7^ CFU/mL (MOI = 1:5), and the expressions were dose-dependently increased, resulting in the most PD-L1^+^ cells in the culture stimulated with 5.0 × 10^8^ CFU/mL (MOI = 1:50) of HK-SA (Figure 3B). Next, we investigated the bioactivities of the structural components of MRSA in neutrophil PD-L1 upregulation. We found that CWE dramatically increased PD-L1 expression in neutrophils with the frequency of PD-L1^+^ neutrophils exceeding 50% at the highest dose (10 µg/mL). CPE also increased PD-L1 expression in neutrophils, although a lesser extent than the CWE. Interestingly, LP isolated from CPE increased the frequencies of PD-L1^+^ neutrophils even at concentrations one-tenth that of CPE. In contrast, nucleic acids did not increase PD-L1 expression in neutrophils (Figure 3C).

Previous publications have described the crucial roles of TLRs in *S. aureus* recognition [13,15]. Therefore, we decided to investigate whether TLRs and their downstream factor, myeloid differentiation factor 88 (Myd88), contribute to PD-L1 upregulation in neutrophils. To determine the TLRs responsible for neutrophil PD-L1 upregulation, we first analyzed the mRNA expressions of TLRs in the HK-SA stimulated neutrophils. The mRNA expressions of TLR2 and TLR4, which express the cell surface, were predominantly increased in the HK-SA-exposed neutrophils (Figure 3D,E). Other cell surface receptors, including the TLR1 and TLR6 mRNA levels, were equivalent between the control and the HK-SA-exposed neutrophils. Additionally, intracellular TLRs, such as TLR3, TLR7, TLR8, and TLR9, also had similar expressions between the control and HK-SA-exposed neutrophils (Figure S2). The mRNA expression of MyD88, a universal adaptor molecule predominantly involved in TLR signal transduction, except for TLR3, also increased in the HK-SA-stimulated neutrophils (Figure 3F).

Following the RNA expression profiles, we performed stimulation assays using neutrophils isolated from WT, TLR2-KO, TLR4-KO, and MyD88-KO mice with HK-SA, CWE, and LP stimulation. In the HK-SA-stimulated neutrophils, the upregulation of PD-L1 expression in the WT neutrophils was completely abolished in TLR2 and TLR4-deficient cells. Additionally, the deficiency of MyD88 also impaired PD-L1 upregulation in HK-SA stimulation (Figure 3G). TLR and MyD88 deficiency also suppressed PD-L1 upregulation in the CWE-stimulated neutrophils (Figure 3H). However, only TLR2 and MyD88 deficiency, but not TLR4 deficiency, abolished PD-L1 upregulation in the LP-stimulated neutrophils (Figure 3I). Therefore, *S. aureus* directly triggers PD-L1 upregulation through TLR signaling in neutrophils.

### 3.4. PD-L1^+^ Neutrophils Contribute Not Only to Enhancing Bacterial Clearance but Also to Inflammatory Resolution

**Figure 4:**
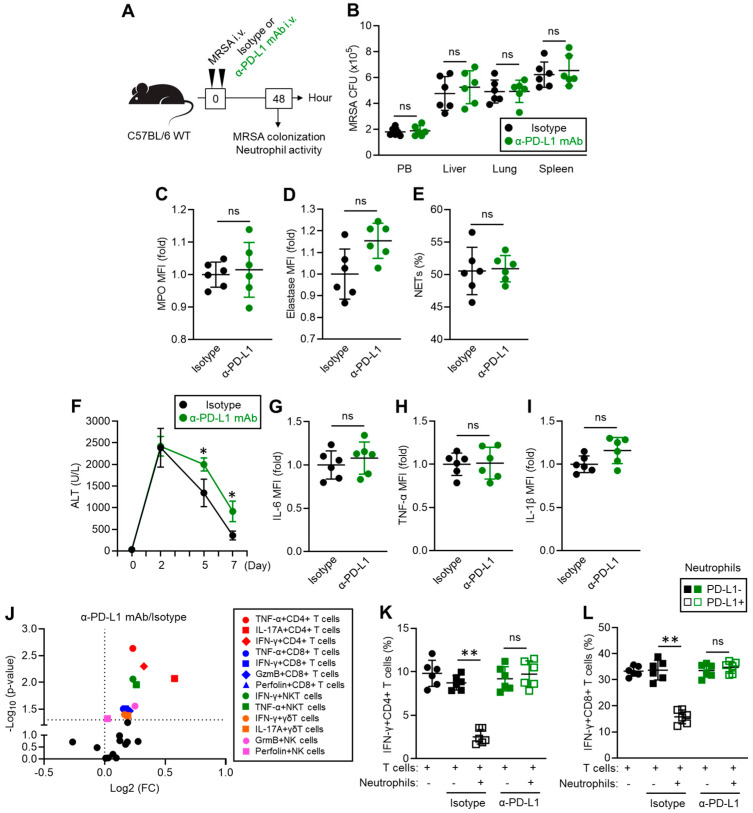
PD-L1 blockade does not compromise neutrophil anti-bacterial immunity; however, it delays the resolution of inflammation in MRSA-infected mice. (**A**) Experimental design of MRSA challenge and anti-PD-L1 mAb administration. Mice were infected with MRSA and received isotype or anti-PD-L1 mAb administration. The bacterial colonization and immune functions of neutrophils were analyzed in the mice at 48 h post-MRSA challenge. (**B**) MRSA colonization in the PB, liver, lung, and spleen in MRSA-challenged mice. The CFUs are indicated as per mL for the PB and per 100 mg for the organs. (**C**–**E**) In vivo neutrophil functional assay. MPO (**C**) and neutrophil elastase (**D**) productions and percentage of NETosis cells (**E**) are shown. (**F**) Plasma ALT concentration in the MRSA-challenged mice. The IL-6 (**G**), TNF-α (**H**), and IL-1β (**I**) production of liver macrophages in the MRSA-challenged mice on day 7. (**J**) Liver leukocyte functional profile of the MRSA-challenged mice. (**K**,**L**) In vitro T cell suppression assay. T cells and neutrophils (PD-L1^−^ or PD-L1^+^) were co-cultured with or without isotype Ab or anti-PD-L1 mAb, and the IFN-γ producing populations of CD4^+^ (**K**) and CD8^+^ (**L**) T cells were analyzed, respectively. The cumulative data are shown as the mean ± standard error (SD) of six samples. Student’s *t*-test was used to analyze the data for significant differences. Values of * *p* < 0.05 and ** *p* < 0.01 are regarded as significant. ns = not significant.

We investigated the immunological role of elevated PD-L1 expression in neutrophils in MRSA infection. Given that PD-L1 has an immunosuppressive role through its interaction with PD-1 expressed in other immune cells, we performed in vivo PD-1/PD-L1 blockade to investigate the influence of abolishing their suppressive effect in MRSA-infected mice [16]. The mice were infected with MRSA via i.v. injection, and either an isotype or anti-PD-L1 mAb was concurrently administered by i.v. injection. After 48 h of MRSA infection, the surviving mice were sacrificed, and MRSA colonization was measured in the peripheral blood (PB), liver, lung, and spleen. Additionally, liver neutrophil function was characterized in each mouse (Figure 4A). PD-1/PD-L1 blockade did not alter MRSA colonization in the mice. All samples showed similar MRSA colony-forming units (CFUs) between the mice injected with the isotype antibody and the anti-PD-L1 mAb (Figure 4B). Application of the anti-PD-L1 mAb did not change the MPO and NE productions in the neutrophils (Figure 4C,D). Additionally, the frequencies of the NETotic cells were equivalent between the mice treated with the isotype antibody and those treated with the anti-PD-L1 mAb (Figure 4E).

Although PD-L1 blockade did not alter the antibacterial activity of the PD-L1^+^ neutrophils, we found that inflammation resolution was delayed by PD-L1 blockade in MRSA-challenged mice. The ALT level, which is an inflammatory parameter in the liver [17], was dramatically increased in the mice 48 h (day 2) post-MRSA challenge, and the levels were comparable between the two groups. The ALT levels gradually decreased in both groups of mice; however, the anti-PD-L1 mAb treatment showed significantly higher levels of ALT at day 5 and day 7 post-MRSA challenge compared to the isotype antibody treatment (Figure 4F). To understand the cause of this delayed inflammation resolution by PD-L1 blockade, we investigated the activity of the other immune cells in the liver involved in the inflammatory response. Inflammatory cytokine production in the macrophages were at equal levels with or without PD-1/PD-L1 blockade (Figure 4G–I), while the inflammatory responses in some types of leukocytes were significantly upregulated by PD-L1 blockade. Pro-inflammatory cytokines and cytotoxic mediator-producing effector CD4^+^ and CD8^+^ T cells were significantly increased in the MRSA-challenged mice treated with the anti-PD-L1 mAb compared to those treated with the isotype antibody (Figure 4J, red and blue plots). Pro-inflammatory cytokine-producing innate lymphocytes, such as γδT cells and NKT cells, and granzyme B or perforin-producing NK cells were also increased in the mice subjected to PD-L1 blockade (Figure 4J, green, orange, and pink plots). To demonstrate the suppressive function of PD-L1^+^ neutrophils, we performed in vitro co-culture of T cells and neutrophils. PD-L1- and PD-L1^+^ neutrophils were obtained as described in Figure 2F, and the cells were co-cultured with splenic CD4^+^ T cells or CD8^+^ T cells with T cell receptor (TCR) stimulation. The cultures were also treated with isotype antibody or anti-PD-L1 mAb. In the presence of the PD-L1^+^ neutrophils, IFN-γ producing CD4^+^ and CD8^+^ T cells were significantly decreased in the cultures compared to those without neutrophils. The PD-L1^−^ neutrophils did not suppress cytokine production in either the CD4^+^ or CD8^+^ T cells, while PD-1/PD-L1 blockade rescued IFN-γ production in the CD4^+^ and CD8^+^ T cells in the presence of the PD-L1^+^ neutrophils (Figure 4K,L). Therefore, PD-L1 mAb administration does not regulate the antibacterial activity of neutrophils; however, it interferes with the inflammatory resolution by sustaining the inflammatory response of leukocytes in MRSA-challenged mice.

## 4. Discussion

Functional suppression based on PD-1/PD-L1 interaction was originally found in anti-tumor immunity, particularly between PD-L1-expressing tumor cells and PD-1-upregulated CD8^+^ T cells, impairing the function of CD8^+^ T cells [16,18]. Recent studies have revealed that PD-L1 expression is widely conserved in myeloid lineage cells, and the protein also interacts with PD-1 expressed on T cells to suppress their activity. This finding has resulted in targeting dendritic cells (DCs) and macrophages for PD-L1 blockade to recover anti-tumor immunity [19,20], while the study of PD-L1^+^ neutrophils is still a minor field and has far less accumulated evidence than other myeloid cells. Additionally, there are some possibilities that PD-L1 may have unknown roles distinct from its general immunosuppressive role in cancer. In fact, a previous report documented another function of PD-L1 as stabilizing neutrophil extracellular trap (NET) formation [21]. Given that neutrophils are abundantly circulating in our bloodstream and frequently involved in diverse immune responses, we decided to investigate the role of PD-L1^+^ neutrophils in infectious diseases.

An interesting finding in our results is the functional difference between PD-L1^−^ and PD-L1^+^ neutrophils in MRSA infection. It is clear that PD-L1^+^ neutrophils possess significantly enhanced anti-bacterial activity compared to PD-L1^−^ neutrophils. The increased population of PD-L1^+^ neutrophils in MRSA-infected mice suggests that this population may predominantly combat bacteria during infection. This evidence supports the concept that the neutrophil population is heterogeneous, and each population has a specific role in various conditions, including infection [22]. However, we have not elucidated the mechanism by which neutrophils control PD-L1 expression in their population. We found that MRSA cell wall extract (CWE) and lipoproteins (LP) triggered PD-L1 expression through Toll-like receptors (TLRs); however, this response spontaneously occurred in the neutrophils of MRSA-challenged mice. Therefore, this finding cannot explain the selective upregulation of PD-L1 expression in neutrophils. Indeed, it is challenging to elucidate the PD-L1 upregulation mechanism under physiological conditions, as various factors may influence neutrophil characteristics. Additionally, we have not explored the mechanistic aspects of how PD-L1 expression enhances neutrophil function against MRSA invasion. A previous report revealed that PD-L1 expression prevents apoptosis and extends their survival in human neutrophils [23]. If this functional modification also occurs in MRSA infection, it might be a possible mechanistic change that prolongs the survival of neutrophils and maintains their effective function against pathogens. A recent finding suggests that PD-L1 can act as a transcription factor [24], raising the possibility that neutrophils also possess a PD-L1-mediated transcriptional regulation mechanism in immune activity. Further investigation is required to elucidate whether increased PD-L1 expression is merely a marker or serves a specific function in neutrophils.

As another new insight into the role of PD-L1^+^ neutrophils in MRSA infection, we propose their supportive role in inflammatory resolution by suppressing the inflammatory activity of leukocytes via PD-1/PD-L1 interaction. Our data suggest that inflammatory leukocytes were increased in the liver of MRSA-challenged mice upon the administration of anti-PD-L1 mAb. PD-L1 expression was predominantly elevated in the neutrophils of the livers of MRSA-infected mice compared to those under the naïve condition; therefore, we suspect that these cells play a suppressive role against inflammatory leukocytes. We also provide strong evidence of PD-L1^+^ neutrophil-mediated suppressive effects through in vitro neutrophil and T cell co-culture. In summary, blocking PD-L1 on neutrophils impaired the indispensable negative regulation of leukocyte inflammatory responses in MRSA-infected livers, which consequently delayed inflammatory resolution. This represents a new understanding of the role of PD-L1^+^ neutrophils in regulating excessive inflammation during infectious diseases. Although our results strongly support the suppressive role of PD-L1^+^ neutrophils against leukocytes, our study has some limitations. We did not perform neutrophil depletion to prove the importance of their suppressive role against leukocytes. Since neutrophil depletion simply accelerates MRSA colonization during the acute phase, we were unable to take this approach. One possibility is to use neutrophil-specific PD-L1 knockout (KO) mice, which can be created by crossing *Pdl1*-floxed mice and S100 calcium-binding protein A8 (calgranulin A)-Cre mice [25,26]. Additionally, PD-L1 expression in other cells may need to be characterized more deeply under MRSA infection. For instance, other myeloid cells, such as DCs, macrophages, and hepatocytes, might express PD-L1 in MRSA-infected mice by excessive inflammation. Since neutrophils frequently circulate in the bloodstream and reside in tissues, PD-L1^+^ neutrophils may have a higher frequency of contact with leukocytes than other PD-L1^+^ cells. However, the background effect of elevated PD-L1 expressions in other cells must be considered.

Recent studies have revealed novel roles and functions of neutrophils that far exceed the traditional understanding. This report also provides new evidence of neutrophil biology. Since the evidence is still accumulating and some findings are contradictory, immunotherapy targeting neutrophils has not yet been applied clinically. However, summarizing solid findings may support the creation of new strategies for neutrophil-based therapy, similar to targeting T cells, in the future.

## 5. Conclusions

**Figure 5:**
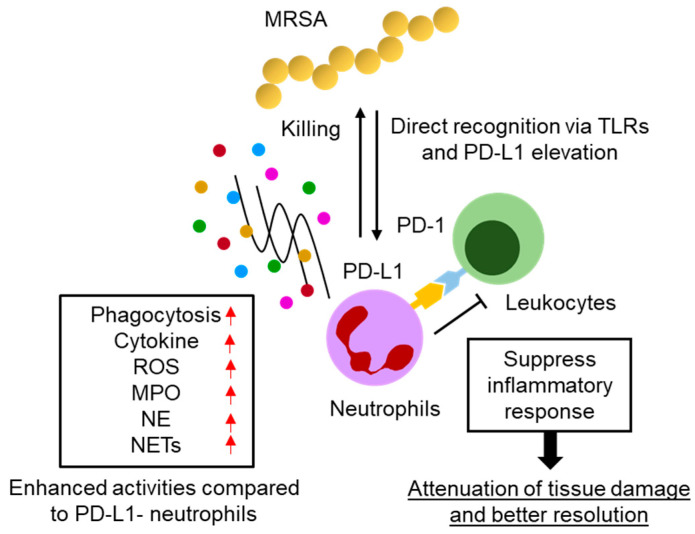
Hypothetical diagram of PD-L1^+^ neutrophil function in the acute phase of MRSA infection.

The results of this study are summarized in the diagram represented in Figure 5. Under MRSA infection, PD-L1 expression is upregulated in neutrophils by the recognition of bacterial structural components through extra-cellular TLRs. The PD-L1^+^ neutrophils are more highly reactive than PD-L1^−^ neutrophils, and their populations effectively eliminate MRSA by enhanced phagocytic activity and increase the productions of cytokine, ROS, MPO, NE, and NETs. On the other hand, PD-L1^+^ neutrophils cease the excessive tissue inflammation associated with accumulating inflammatory leukocytes through PD-1/PD-L1 interaction, resulting in supporting inflammatory resolution.

## Data Availability

The original data presented in the study was represented in the Supplementary Materials. Further inquiries can be directed to the corresponding authors.

## Supplementary Materials

The following supporting information can be downloaded at: https://www.mdpi.com/article/10.3390/pathogens13050401/s1, Figure S1: PD-L1 mRNA expression in peripheral blood neutrophils. Figure S2: Gene expression of TLR in neutrophils. Table S1: Primer sequences for real-time PCR.
