# Supplementary material for: Neutrophils Expressing Programmed Death-Ligand 1 Play an Indispensable Role in Effective Bacterial Elimination and Resolving Inflammation in Methicillin-Resistant Staphylococcus aureus Infection
Source: Pathogens. 2024 May 11;13(5):401. doi: 10.3390/pathogens13050401 (PMC11124513; doi:10.3390/pathogens13050401)
Supplement: Supplementary file 1 [file pathogens-13-00401-s001.zip › pathogens-2962127-supplementary.pdf]

# Supplemental Material

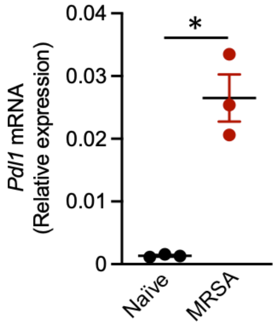


**Supplemental Figure S1.** PD-L1 mRNA expression in peripheral blood neutrophils. Peripheral blood neutrophils were isolated from naïve or MRSA infected mice (48 h), then total RNA isolated from the cells were subjected to real-time PCR for quantification of PD-L1 mRNA expression. The cumulative data were shown as mean ± standard error (SD) of three samples. Student t-test was used to analyze data for significant difference. Values of *$p$ < 0.001 was regarded as significant.

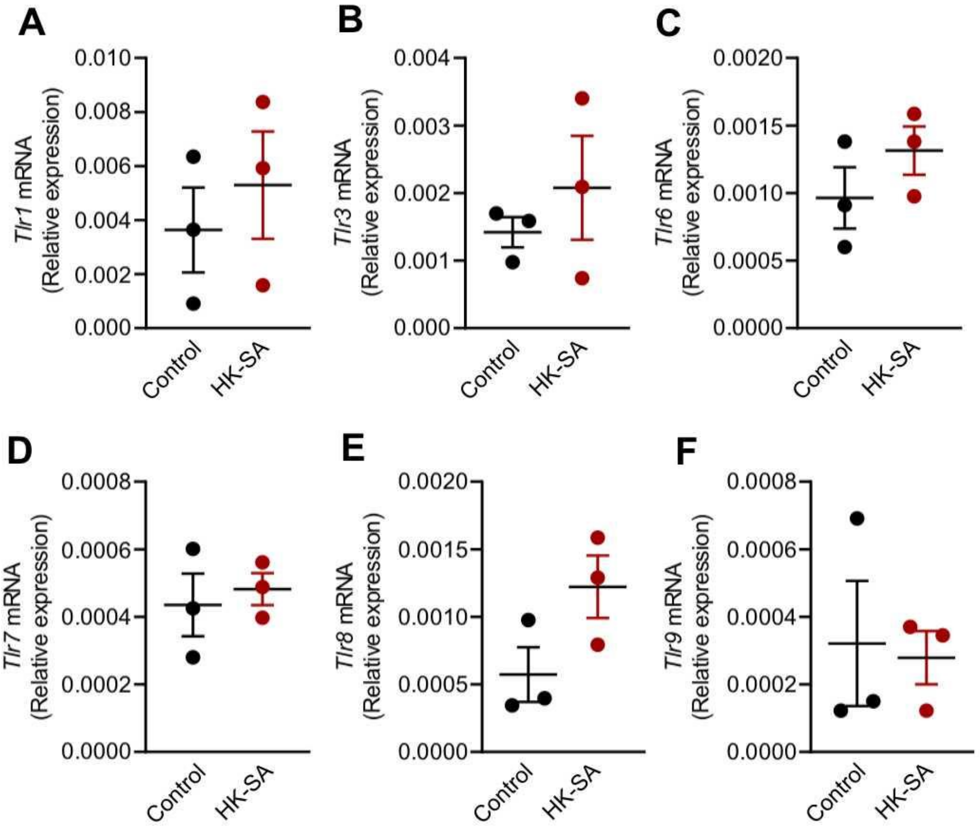


**Supplemental Figure S2.** Gene expression of TLR in neutrophils. Neutrophils were cultured with control or HK-SA (MOI=1:10) at 37°C for 3 h, then isolated total RNA was subjected to real-time PCR. The mRNA expression of TLR1 (A), TLR3 (B), TLR6 (C), TLR7 (D), TLR8 (E) and TLR9 (F) are shown. The cumulative data were shown as mean ± standard error (SD) of three samples. Student t-test was used to analyze data for significant differences.

**Supplemental Table S1.** Primer sequences for real-time PCR

*Pdl1*-forward: 5′- GCTCCAAAGGACTTGTACGTG-3′

*Pdl1*-reverse: 5′-TGATCTGAAGGGCAGCATTTC-3′

*Tlr1*-forward: 5′-TGAGGGTCCTGATAATGTCCTAC-3′

*Tlr1*-reverse: 5′-AGAGGTCCAAATGCTTGAGGC-3′ *Tlr2*-forward: 5′-GCAAACGCTGTTCTGCTCAG-3′

*Tlr2*-reverse: 5′-AGGCGTCTCCCTCTATTGTATT-3′ *Tlr3*-forward: 5'-GTGAGATACAACGTAGCTGACTG-3' *Tlr3*-reverse: 5'-TCCTGCATCCAAGATAGCAAGT-3' *Tlr4*-forward: 5′-AGGCACATGCTCTAGCACTAA-3′ *Tlr4*-reverse: 5′-AGGCTCCCCAGTTTAACTCTG-3′

*Tlr6*-forward: 5′-TGAGCCAAGACAGAAAACCCA-3′

*Tlr6*-reverse: 5′-GGGACATGAGTAAGGTTCCTGTT-3′

*Tlr7*-forward: 5'-ATGTGGACACGGAAGAGACAA-3'

Tlr7-reverse: 5'-GGTAAGGGTAAGATTGGTGGTG-3'

*Tlr8*-forward: 5'-GAAAACATGCCCCCTCAGTCA-3'

*Tlr8*-reverse: 5'-CGTCACAAGGATAGCTTCTGGAA-3'

*Tlr9*-forward: 5'-ATGGTTCTCCGTCGAAGGACT-3'

*Tlr9*-reverse: 5'-GAGGCTTCAGCTCACAGGG-3'

*Gapdh*-forward: 5′-TGTGTCCGTCGTGGATCTGA-3′

*Gapdh*-reverse: 5′-TTGCTGTTGAAGTCGCAGGAG-3′
